# In vitro larval rearing method of eusocial bumblebee *Bombus terrestris* for toxicity test

**DOI:** 10.1038/s41598-022-19965-0

**Published:** 2022-09-22

**Authors:** Yuto Kato, Shingo Kikuta, Seth M. Barribeau, Maki N. Inoue

**Affiliations:** 1 Department of Agriculture, Tokyo University of Agriculture and Technology, 3-5-8 Saiwai-cho, Fuchu, Tokyo 183-8509 Japan; 2 College of Agriculture, Ibaraki University, Ami, Ibaraki 300-0393 Japan; 3 Institute of Infection, Veterinary, and Ecological Sciences, The University of Liverpool, Liverpool, L69 7ZB UK

**Keywords:** Ecology, Environmental sciences

## Abstract

Bumblebees are important pollinators of wild and agricultural plants but recently have been declining due to various stressors, such as pesticides and diseases. Because of the haplo-diploid sex determination system in hymenopterans, experiments using micro-colonies (small sub colonies without a queen) to identify risks to bumblebee health are limited as they are only able to produce males. Therefore, an experimental protocol for rearing bumblebee larvae in vitro is needed to better understand effects on worker larvae. Here, we aimed to establish a rearing method for larvae of *Bombus terrestris* for use in risk assessment assays. To confirm the validity of our rearing method, we tested two insecticides used for tomato cultivation, chlorfenapyr and dinotefuran. *Bombus terrestris* larvae fed with a high nutrient quantity and quality diet increased growth per day. All chlorfenapyr-exposed individuals died within 10 days at 2000-fold dilution, an application dose used for tomatoes. There were significant differences in adult emergence rate among almost all chlorfenapyr treatments. On the other hand, sublethal dinotefuran-exposure did not affect rates of pupation and adult emergence, growth, or larval and pupal periods. Although larvae were smaller than in the natural colony, this rearing method for *B. terrestris* larvae proved to be effective at evaluating realistic sub-colonies to pesticide exposures.

## Introduction

Pollinators play an important role in crop production and wild plant reproduction^1,2^. The economic value of these pollinators for global crop production was estimated at €15.3 billion in 2005, and the productions of vegetables, fruits, and nuts are highly dependent on pollinators^3,4^. In recent years, however, declines in pollinators, especially bee pollinators, have been reported worldwide, and these declines are thought to be due to a combination of factors, including pesticides, climate change, habitat loss, and parasites^2,5^.

Bumblebees (Apidae: Hymenoptera) are eusocial insects with an annual colony cycle. Queens emerge from hibernation in the spring and establish new colonies, each producing hundreds of workers during the summer. Since their commercialization, they are mainly used for pollination of Solanaceae plants in greenhouses because of their unique vibration pollination method. Wild bumblebee population declines have been reported mainly in Europe and the United States; four bumblebee species in Europe have disappeared from 11 countries in the last 60 years^6^; in the United States, the prevalence of the parasite *Nosema bombi* was high in bumblebee species whose populations are declining^7,8^.

Pesticides and other agricultural chemicals are also important factors responsible for bumblebee decline. For bumblebees, even low-level exposures of pesticide have a variety of sub-lethal effects, including delayed colony development, reduced queen ovary development and egg-laying, as well as reduced learning, memory, and pollination abilities^9–13^. Pollinators are most often indirectly exposed to pesticide residues on crops, so it is likely that both adults and larvae in colonies are exposed to pesticides through their diet^14^. Given that bumblebees are at risk of being exposed to pesticides throughout their life history, it is important to evaluate the effects on larvae as well as adults.

Thus far, risk assessment studies in bumblebees have been conducted using micro-colonies^13,15,16^, which contain only a few workers. Toxicity tests using micro-colonies have advantages due to its ease of repetition and standardization of data, and thus the European Food Safety Authority (EFSA) recommends the use of micro-colonies for risk assessments of bumblebees^17^. However, the protocols of the previous studies differed in the number of workers, the number of days after worker hatching, and the timing of the end of the experiment^18–21^. The data cannot be easily compared because the amount of food consumed by adults and larvae cannot be accurately measured. In addition, workers do not mate, so only males are produced, and it is not possible to evaluate the effects of the assessed risk factor on larvae destined to be workers or queens. Therefore, it is essential to establish an experimental protocol that more closely resembles a natural colony, where diet and feeding can be controlled, in other to evaluate the effects of experimental treatments on worker and queen larvae as well as male larvae.

However, larvae of social insects are much more difficult to rear artificially than those of non-social insects because they grow under the constant care of workers. Although the method for the larval rearing of honeybees is already standardized^22^, there is little knowledge of larval rearing in bumblebees^13,23^ or stingless bees^24,25^. While they belong to the same family, larval rearing conditions in the colony are quite different, making it difficult to simply apply the rearing systems established for honeybees to bumblebees.

**Figure 1:**
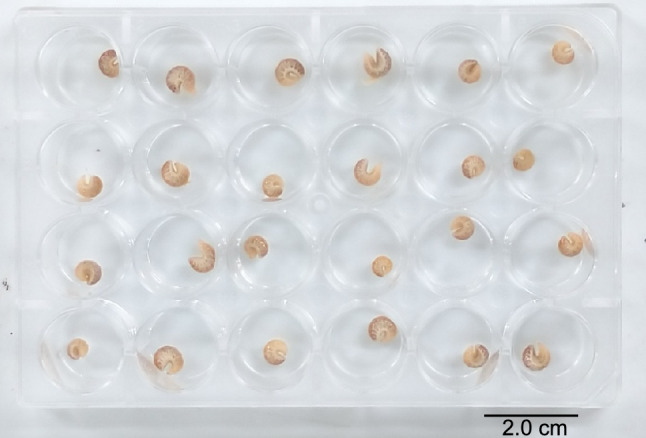
Rearing of bumblebee larvae in a 24-well plate.

Here, we aimed to establish a rearing method for bumblebee larvae for use in risk assessment assays (Fig. 1). *Bombus terrestris* was used in this study because it has been commercialized with a large colony size and has been used in many previous studies. To confirm the validity of our rearing method, we tested two insecticide formulations with different mechanisms of action used in tomato cultivation. We selected chlorfenapyr and dinotefuran: both are used not only for tomatoes, where bumblebees are used for pollination, but also for a variety of crops grown in open fields. Chlorfenapyr, a pyrrole insecticide, exhibits broad-spectrum insecticidal activity and targets the mitochondrial ATP synthesis^26^, and dinotefuran, a neonicotinoid insecticide^27^, also has high insecticidal activity and affects a broad spectrum of insects.

## Results

### Effect of feeding frequency on *B. terrestris* larvae

**Figure 2:**
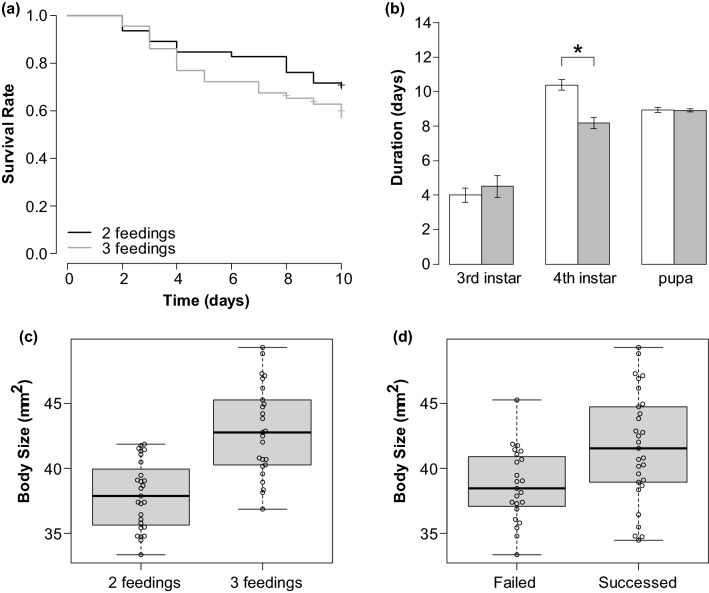
Effect of feeding frequency on *B. terrestris* larvae. (**a**) Cumulative survival of bumblebee larvae during 10 days after each treatment of the different feeding frequency. No significant differences were detected among the treatment groups. (**b**) The duration of each growth stage, 3rd, 4th, and pupal periods, at different feeding frequencies. White bar indicated the two feeding frequency group; the gray bar, the three feeding frequency group. Asterisks indicate the significant difference. (**c**,**d**) Effect of feeding frequency on (**c**) the pre-pupal size of each treatment and (**d**) the pre-pupal size of individuals that emerged and did not emerge. Points on plots indicate each sample.

**Figure 3:**
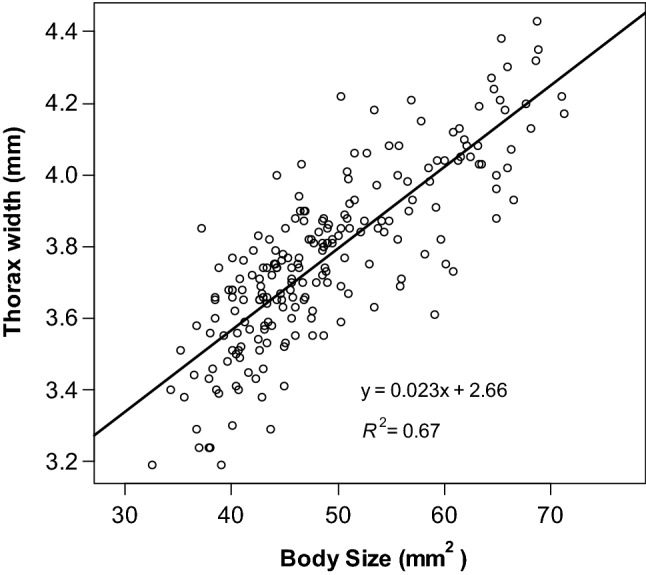
Scatter plot of larval size before pupation and thorax width after hatching. The line represents the regression line.

There was no significant difference between two- and three-feeding groups in survival time (Log-rank test: χ^2^ = 0.2, *F* = 1, *P* = 0.6, Fig. 2a) and pupation rate (58.7% in the two-feeding group and 55.8% in the three-feeding group; χ^2^ test: χ^2^ = 0.07, *F* = 1, *P* = 0.79), but the adult emergence rate was significantly higher in the three-feeding group (17.4% in the two-feeding group and 48.8% in the three-feeding group; Fisher's exact test: *P* = 0.003). The larval duration was significantly shorter in the three-feeding group only in 4th instar (Wilcoxon rank sum test: 3rd instar, *W* = 54, *P* = 0.42; 4th instar, *W* = 541, *P* < 0.01; pupa, *W* = 85.5, *P* = 0.96, Fig. 2b). The larval size was also significantly larger as the feeding frequency was increased (Wilcoxon rank sum test: *W* = 93, *P* < 0.01, Fig. 2c). The pre-pupal larval sizes of all examined individuals were significantly larger than the successfully emerged larvae than the unsuccessfully emerged larvae (Wilcoxon’s rank sum test: *W* = 195, *P* = 0.01, Fig. 2d). There was also a strong correlation between larval size and thorax width immediately after defecation before pupation (Pearson correlation analysis: *T* = 20.511, *F* = 210, *P* < 0.01, *r* = 0.82, Fig. 3).

### Effect of different diet nutrients on *B. terrestris* larvae

**Figure 4:**
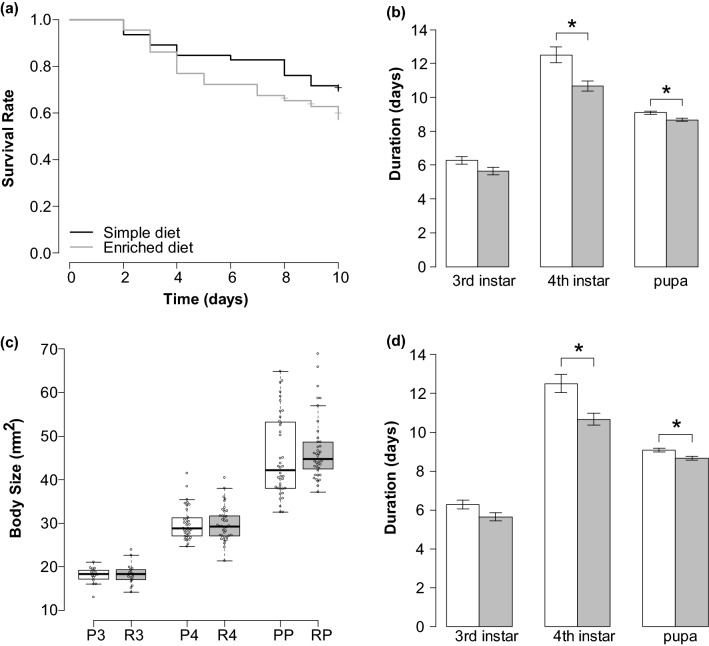
Effect of different diet nutrients on *B. terrestris* larvae. (**a**) Cumulative survival of *B. terrestris* larvae during 10 days after each treatment of the different feeding frequency. No significant differences were detected among the treatment groups. (**b**) The duration of each growth stage, 3rd, 4th, and pupal periods, at different diet nutrients. White bar indicated the simple diet group; the gray bar, the enriched diet group. Asterisks indicate the significant difference. (**c**,**d**) Effect of diet nutrients (**c**) larval size at the 3rd and 4th instar molting and before pupation and (**d**) growth per day at the 3rd and 4th instar. Points on plots indicate each sample. Error bars represent the standard error range, and asterisks indicate the significant difference.

The survival rate did not differ significantly between the enriched diet group and the simple diet group (Log-rank test: χ2 = 1.8, *F* = 1, *P* = 0.2, Fig. 4a). There was no significant difference in growth rate at 3rd instar, but growth rate at 4th instar was significantly greater in the enriched diet group (*t* test: 3rd instar, *t* = -1.58, *F* = 20, *P* = 0.13; 4th instar, *t* = -3.49, *F* = 69, *P* < 0.01, Fig. 4b). The duration of 4th instar and pupae was significantly shorter in the enriched diet group (Wilcoxon rank sum test: 3rd instar, *W* = 86.5, *P* = 0.081; 4th instar, *W* = 796, *P* < 0.01; pupae, *W* = 729, *P* < 0.01, Fig. 4c). There were no significant differences among the treatments in larval size at molting and pre-pupation (Wilcoxon rank sum test: 3rd instar, *W* = 138, *P* = 0.95; 4th instar, *W* = 644, *P* = 0.98; pre-pupation, *W* = 609.5, *P* = 0.095, Fig. 4d), pupation rate (80.4% for simple diet, 77.1% for enriched diet; χ2 test: χ2 = 0.02, *F* = 1, *P* = 0.88), and the adult emergence rate (67.4% for simple diet, 66.7% for enriched diet; χ2 = 0, *F* = 1, *P* = 1).

### Effect of chlorfenapyr-inoculation of on *B. terrestris* larvae

**Figure 5:**
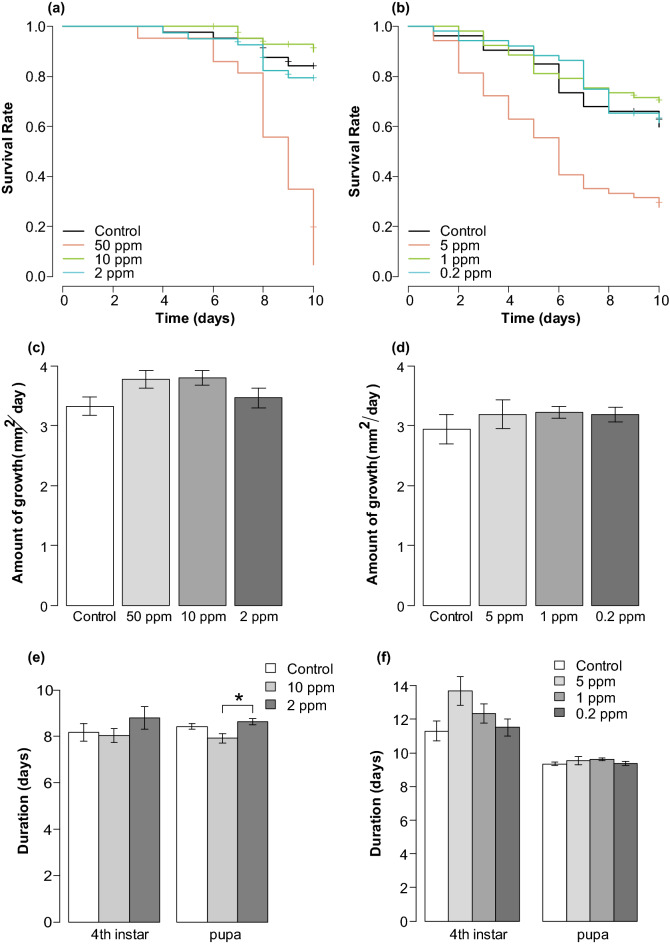
Cumulative survival of *B. terrestris* larvae during 10 days after each treatment in the oral inoculation test of (**a**) chlorfenapyr and (**b**) dinotefuran. Growth rate during one day after molting at the 4th instar for each treatment of the oral inoculation test of (**c**) chlorfenapyr and (**d**) dinotefuran. The durations of 4th instar and pupae in the oral inoculation test of (**e**) chlorfenapyr and (**f**) dinotefuran. Error bars indicate standard errors, and asterisks indicate the significant difference.

The survival time was significantly different between control and the 50 ppm treatment (Log-rank test: *P* < 0.01) but not between control and 10 ppm treatment (*P* = 0.6), 2 ppm (*P* = 0.6) (Fig. 5a). Larval growth rate per day of 4th instar did not differ significantly among any combinations of treatments (Table 1, Fig. 5c). The pupation rate was significantly lower only in the 50 ppm treatment than all treatments (4.7% in 50 ppm, 90.9% in 10 ppm, 80.5% in 2 ppm, and 83.7% in control), while significant differences were detected in adult emergence rate among almost all treatments, and the higher the dilution was, the higher the adult emergence rate tended to be (0.0% in 50 ppm, 15.9% in 10 ppm, 61.0% in 2 ppm, and 81.4% in control), except for the comparison of adult emergence rate between control and the 2 ppm treatments with slightly statistically insignificant (Table 1). The 4th instar and pupal duration of each treatment was significantly different only in the comparison of pupal duration between 10 and 2 ppm treatments, where the pupal duration was longer in the 2 ppm treatment (Fig. 5e, Table 1).

**Table 1:** All groups comparison of the 4th instar period, pupal period, growth per day of 4th instar, pupation rate, and emerging rate in the oral inoculation test of chlorfenapyr.

Treatments	4th instar period				Pupal period				Growth rate per day of 4th instar				Pupation rate	Emergence rate
	Tukey–Kramer test				Tukey–Kramer test				Tukey–Kramer test				Fisher's exact test	
	diff	lwr	upr	*P*	diff	lwr	upr	*P*	diff	lwr	upr	*P*	*P*	*P*
Control vs. 50 ppm									0.449	− 0.138	1.036	0.195	** < 0.01**	** < 0.01**
Control vs. 10 ppm	− 0.120	− 1.450	1.211	0.975	− 0.500	− 1.158	0.158	0.170	0.474	− 0.058	1.001	0.098	0.566	** < 0.01**
Control vs. 2 ppm	0.633	− 0.769	2.035	0.529	0.211	− 0.205	0.627	0.446	0.137	− 0.431	0.704	0.922	0.566	0.053
50 ppm vs. 2 ppm									0.025	− 0.521	0.571	0.999	** < 0.01**	**0.024**
50 ppm vs. 2 ppm									− 0.312	− 0.894	0.269	0.499	** < 0.01**	** < 0.01**
10 ppm vs. 2 ppm	0.753	− 0.511	2.018	0.334	0.711	0.032	1.391	**0.038**	− 0.337	− 0.863	0.188	0.340	0.566	** < 0.01**

Bold indicates statistical significance.

### Effect of dinotefuran-inoculation on *B. terrestris* larvae

Comparing the survival time during the 10 days between the control and pesticide-treatment groups, only the 5 ppm treatment was significantly shorter than the control, but there were no significant differences in the other groups (Log-rank test: vs. 5 ppm, *P* < 0.01; vs. 1 ppm, *P* = 0.6; vs. 0.2 ppm, *P* = 0.8, Fig. 5b). On the other hand, there were no significant differences between the control and each pesticide treatments in the pupation rate (27.8% in 5 ppm, 66.0% in 1 ppm, 55.8% in 0.2 ppm, and 52.8% in the control), adult emergence rate (20.4% in 5 ppm, 50.9% in 1 ppm, 38.5% in the 0.2 ppm, and 28.3% in the control), growth rate per day of the 4th instar (Fig. 5d), and larval and pupal period (Fig. 5f). Comparing among the treatments, there was a significant difference only in the pupation and adult emergence rates between the 5 ppm treatment, the highest concentration, and the 0.2 ppm treatment, the lowest concentration (Fisher's exact test: pupation rate, *P* < 0.01, adult emergence rate *P* < 0.01, Table 2).

**Table 2:** All groups comparison of the 4th instar period, pupal period, growth per day of 4th instar, pupation rate, and emerging rate in the oral inoculation test of dinotefuran.

Treatments	4th instar period				Pupal period		Growth rate per day of 4th instar				Pupation rate	Emergence rate
	Tukey–Kramer test				Steel–Dwass test		Tukey–Kramer test				Fisher's exact test	
	diff	lwr	upr	*P*	*T*	*P*	diff	lwr	upr	*P*	*P*	*P*
Control vs. 5 ppm	2.371	− 0.265	5.007	0.094	0.749	0.877	0.247	− 0.343	0.836	0.694	**0.042**	0.374
Control vs. 1 ppm	1.031	− 1.041	3.102	0.565	1.839	0.255	0.282	− 0.189	0.753	0.403	0.642	0.142
Control vs. 0.2 ppm	0.210	− 1.955	2.376	0.994	0.075	1.000	0.245	− 0.232	0.722	0.540	0.845	0.374
5 ppm vs. 1 ppm	− 1.340	− 3.865	1.185	0.510	0.387	0.980	0.035	− 0.526	0.596	0.998	**0.001**	**0.007**
5 ppm vs. 0.2 ppm	− 2.161	− 4.763	0.442	0.139	0.754	0.875	− 0.002	− 0.568	0.564	1.000	**0.028**	0.219
1 ppm vs. 0.2 ppm	− 0.820	− 2.849	1.209	0.716	1.743	0.301	− 0.037	− 0.479	0.404	0.996	0.642	0.374

Bold indicates statistical significance.

## Discussion

Here we show that an increasing the quantity and quality of nutrients given to *B. terrestris* larva resulted in an increase in larval growth. In the oral pesticide inoculation experiments, all chlorfenapyr-exposed individuals were killed within 10 days at 50 ppm, which is the same application concentration used for tomatoes. There was no significant difference in the survival rate during the larval period at lower concentrations. The cause of this lack of effect is unclear but could be the result of a nonlinear response to the compound with low concentrations having limited effect below a critical threshold, or possibly faster degradation of active ingredient at low dose. Dinotefuran-inoculation experiments resulted in shorter survival time and lower pupation rate in the high concentration groups, but no significant effects were observed in lower exposure doses compared to the control. The larval size at pre-pupation was strongly correlated with the adult thorax width, suggesting a very useful indicator for larval growth.

In this study, the larvae that were fed more frequently were more likely to emerge than larvae that fed less. Furthermore, those larvae that did emerge were larger than those that eventually failed to emerge. In addition, the larvae fed with only dry pollen and sugar water took longer to grow to the same size as those fed with additional amino acids and minerals. These results suggested that even in bumblebees with a wide variety of adult sizes within a colony, there was a threshold of larval size required for pupation and metamorphosis into adulthood. Food availability plays a crucial role in the regulation and duration of larval development and the timing of subsequent developmental events, and the alteration in the duration of growth under starvation conditions has been observed in many insect species^28,29^. The tobacco hornworm *Manduca sexta* do not molt or pupate until they have grown to a certain “critical weight”, by extending the larval period, when starved^30^. The prolonged larval period by undernourishment exhibited by *B. terrestris* appears similar to the response of *M. sexta*. Bumblebee larvae are fed by the adults, and thus extending the larval period may increase the chance that they will be able to obtain sufficient food. In a previous study by Cnaani et al. (2000)^31^, larval durations were about 2 days for the 3rd instar, 4 days for the 4th instar, and 14 days from the 5th instar to emergence. Considering the reduction of the larval period in increasing food availability (Fig. 4), the prolonged larval period suggested that the nutrient availability in our artificial rearing method was lower than that obtained within the colony. In fact, the thorax width of the adults in this study ranged from 3.19 to 4.43 mm, while the adults in the natural colony ranged from 2.3 to 6.9 mm^32^. Furthermore, the survival rate of the control treatment of the dinotefuran-inoculation test (60.4%) was lower than chlorfenapyr-inoculation test (86.1%). This may be because one of the colonies used for the experiments was not likely to be in good condition with small number of larvae, suggesting that colony condition also affect larval growth. While this suggests we have opportunity to improve rearing protocols, this approach represents a very promising method for more realistic bumblebee bioassays.

In the oral inoculation test of chlorfenapyr, the pupal period was shorter in the 10 ppm treatment than in the 2 ppm treatment, probably due to lower emergence rate (Table 2); other pesticide treatments did not show significant differences between control. Furthermore, the larvae size before pupation was not significantly different between control and the pesticide treatments except for the 50 ppm treatment, but the adult emergence rate in all pesticide treatments was significantly lower than that in control. This result suggested that the chlorfenapyr may negatively affect larvae or pupae during the processes for adult emergence. Since mortality during the pupal period was also observed in the twice-fed treatment, nutrients required for adult emergence may not be sufficient due to chlorfenapyr treatments. Considering the fact that chlorfenapyr uncouples oxidative phosphorylation in the mitochondria of bumblebee adults^33^, which leads to the energy loss in the cells^34^, it is possible that chlorfenapyr prevents the larvae from preserving enough nutrients for adult emergence.

Chlorfenapyr is classified as "moderately hazardous (Class II)" according to WHO criteria. It takes 21 days to degrade to below detectable concentrations in soil and crops, suggesting that it may remain in the environment for a long time even at low concentrations^35^. Under laboratory conditions, chlorfenapyr is highly toxic to honeybee workers, with exposures not only by direct spraying, but also by feeding of contaminated food and contact with sprayed leaves^36^. Oral inoculation of chlorfenapyr at the recommended concentration killed all treated bumblebee workers within one week^18^. Chlorfenapyr is one of the insecticides used in greenhouses with bumblebee colonies, but few studies have examined its effects on bumblebees. Our experiments showed that chronic exposure to chlorfenapyr can cause larval death during pupation. This loss of larva will likely result in poor colony development and reproductive failure, and ultimately population decline.

The recommended concentration of dinotefuran for tomato crops is 2000-fold dilution (100 ppm), but oral inoculation at this concentration caused acute lethality to bumblebee larvae in this study. Lethal activity was also observed at 5 ppm (40,000-fold), indicating that this pesticide is highly toxic to *B. terrestris* larvae. However, no significant effect was observed below 1 ppm concentration compared to the control. Since *B. terrestris* adults can clear neonicotinoid imidacloprid at low concentrations^37^, the dinotefuran-treated larvae may also clear (detoxified or metabolized) at low concentrations below 1 ppm. In the previous study, field-realistic dose exposure of imidacloprid to bumblebee adults reduce humoral antimicrobial activity and phenoloxidase activity^38^, and chronic exposure to neonicotinoid pesticide increased the expression of detoxification genes^39^ and antimicrobial peptide genes^40^. If exposure to dinotefuran even at low concentrations that can be cleared from the body increases bee susceptibility to other insecticides and parasites, this formulation may pose a serious threat to bumblebees. While dinotefuran is the most widely used neonicotinoid insecticide in Japan, since it is not registered as a pesticide in Europe, it has not been studied for its effects on sub-lethal effects on bumblebees. Therefore, we urgently need to investigate the effects of exposure to sublethal concentrations of dinotefuran on the immune system of the larvae, or on the expression of immune and detoxification genes, and the effects of simultaneous exposure to different insecticides and parasites.

In this study, a rearing method for *B. terrestris* larvae using simple diet was established and proved to be effective to evaluate the stressors, such as pesticides. The establishment of an artificial rearing method for honey bee larvae has been hugely valuable; allowing us to evaluate the effects of not only neonicotinoid insecticides and parasites alone, but also the effects of simultaneous exposure to parasites and pesticides^41–43^, and other approaches such as metabolomics^44^. Since each bumblebee species has different ecological characteristics, it may be necessary to modify rearing methods for other species other than *B. terrestris*. Still, this rearing method of bumblebees is expected to be applied in various fields to elucidate and solve the causes of population decline.

## Materials and methods

### The rearing method of *Bombus terrestris* larvae

#### Insect

We used five commercial colonies of naïve bumblebees, *B. terrestris* (Natupol, Arysta Life Science, Tokyo); one colony was used in each of the three experiments, and two colonies was used in the inoculation test of dinotefuran due to small number of larvae in one of the colonies. The colonies were placed in a dark room for 3–6 days after arrival. Before the experiments, 16 workers were randomly collected from each colony, and their midguts were examined to confirm parasite-free under light microscope. The colony was anesthetized with carbon dioxide (KENIS LIMITED, Osaka) for 5–10 min, and the larvae were taken out after removal of adults under red light illumination. Larvae of a batch grow in a single cell from the egg stage to the late 3rd instar, and each larva in a private cell from the last instar. For all the experiments in this study, the cell with multiple larvae was removed from the colony. Then, larvae from the cells were individually carefully transferred to 24-well plates using tweezers (Fig. 1) and placed in a plastic container (23 × 13 × 5 cm) with a 120 mL plastic cup of saturated sodium chloride solution to control humidity. They were kept under dark condition at a temperature of 34 °C and a relative humidity of 75%. During feeding, the food was kept at 35 °C and a 24-well plate was placed on a hot plate at 35 °C so that the larvae were not affected by the temperature change. Larvae were fed by placing droplets on their abdomens using a micropipette with an interval of at least 5 h.

#### Effect of the number of feedings on each parameter

Food of 50% sucrose solution containing 40% (w/v) dried pollen with Natupol, 10% (w/v) yeast extract (Becton Dickinson, Tokyo), and 1% (w/v) sodium caseinate (FUJIFILM Wako Pure Chemical Corporation, Osaka) was presented to larva according to their instar; 1 × 2 μl for 1st instar larvae, 2 × 2 μl for 2nd larvae, and 3 × 2 μl for 3rd larvae. We tested how the number of feeding times per day affect growth and eclosion by comparing individuals fed twice daily (N = 46) or three times daily (N = 43). Survival rate for 10 days, larval period, pupation rate, hatching rate, and body size of larvae were compared among treatments. Bumblebee larvae pupate after defecation for several days in the late 4th instar, so their bodies become smaller just before pupation. In this study, larvae within 12 h after the onset of defecation were photographed from above using a phone camera with a ruler (Xperia Ace SO-02L, Sony, Tokyo) and measured using by Image J 1.52a (https://imagej.nih.gov/ij/) as an index of larval size. To confirm the accuracy of this method, we also measured the adult thorax width upon eclosion, which is commonly used as an indicator of adult body size^12,33^.

#### Effect of nutrition on larval growth

We prepared a simple diet consisting of 50% sucrose solution containing 40% (w/v) dried pollen, and an enriched diet of 50% sucrose solution containing 40% (w/v) dried pollen, 10% (w/v) yeast extract, and 1% (w/v) sodium caseinate with nutrients such as amino acids and minerals. We presented 46 individuals with the simple diet and 48 individuals with the enriched diet. Larva were fed three times a day with an interval of at least 5 h. In these two treatments, larval size at 3rd and 4th molting, larval size before pupation, growth per day of 3rd and 4th instar, the duration of each growth stage, the 10-day survival rate, and the pupation and hatching rates were compared.

### Effects of oral inoculation of pesticides on *B. terrestris* larvae

#### Oral inoculation test of chlorfenapyr

The 3rd and 4th instar larvae were fed three times a day at a rate of (larval instar) × 2 µl per feeding. A total of four treatments were prepared: a control and three pesticide treatments. Food was 50% sucrose solution containing 40% (w/v) dried pollen, 10% (w/v) yeast extract, and 1% (w/v) sodium caseinate. For the inoculation, the commercial formulations of chlorfenapyr (Kotetu floorable, Kumiai Chemical Industry Co., Ltd, Tokyo, Japan) was used with 50% sucrose solution diluted 2,000-fold (50 ppm), which is the same concentration used for spraying tomatoes, 10,000-fold (10 ppm), and 50,000-fold (2 ppm). The sample was 43 individuals for the control, 43 individuals for the 50 ppm treatment, 44 individuals for the 10 ppm treatment, and 41 individuals for the 2 ppm treatment. The diets with the pesticide were dispensed in small portions of 500 µl each and stored at 4 ℃. The food was re-prepared every 5 days to reduce the effects of degradation of the active ingredient. Survival rate, pupation rate, hatching rate, duration of each growth stage, and larval growth per day of the fourth instar for 10 days in the treatment sections were compared.

#### Oral inoculation test of dinotefuran

The 2nd and 3rd instar larvae were used for this test. We followed the same treatments and feeding methods as above. Preliminary experiments were conducted on each 24 individual larvae to determine the concentration for oral inoculation with the commercial formulations of dinotefuran (Alvarin granule water solution, Agro-Kanesho Co., Ltd. Tokyo, Japan); dinotefuran was diluted 2000-fold (100 ppm) and 20,000-fold (10 ppm), with 50% sucrose solution, which is the same concentration spraying for tomatoes, but all of treated larvae died within a day at 100 ppm concentration and on the next day at 10 ppm concentration. Then, the dinotefuran of concentrations 40,000-fold (5 ppm), 200,000-fold (1 ppm), and 1,000,000-fold (0.2 ppm) diluted using 50% sucrose solution were used to examine the sublethal effects. The sample was 53 individuals for the control, 54 individuals for the 5 ppm treatment, 53 individuals for the 1 ppm treatment, and 52 individuals for the 0.2 ppm treatment. The survival rate, pupation rate, hatching rate, duration of each growth stage, and larval growth per day of the fourth instar for 10 days in the treatment section were compared.

### Statistical analysis

All statistical analyses were performed using R ver. 4.0.2^45^.

For the tests of the feeding number, nutrient diets, and insecticide-inoculations, the Log-rank tests were used to compare the survival times of the treatments, because the proportional hazards assumption was violated. Individuals that pupated during the 10-day period were treated as censored data. P-values were adjusted by the Benjamini–Hochberg method. Chi-squared χ^2^ tests were used to compare pupation rate for the test of the feeding number, and pupation and emergence rates for the test of nutrient diets. Fisher's exact tests were used to compare emergence rate, larval duration, and larval size for the test of the feeding number, and Fisher's exact test with adjustment of P-values by Benjamini–Hochberg method to pupation and emergence rates for the chlorfenapyr-inoculation test, and emergence rate the dinotefuran-inoculation test. Wilcoxon rank sum tests were used for body size of emerged and non-emerged individuals for the test of the feeding number, and larval size and duration of each growth stage for the test of nutrient diets. To determine the relationship between larval size and thorax width immediately after excretion, we used Pearson correlation analysis. *T*-tests were used to compare growth rate per day of 3rd and 4th instar larvae for the test of nutrient diets. Tukey–Kramer tests were used to compare the growth rate per day of the 4th instar and the duration of each growth stage for the chlorfenapyr- and dinotefuran -inoculation tests. Steel–Dwass test was used for interval comparisons of pupal duration for the dinotefuran-inoculation test.

## Data Availability

The datasets during and/or analyzed during the current study available from the corresponding author on reasonable request.
